# Bringing Cervical Cancer Screening Closer to Women: Feasibility of Artificial Intelligence and Remote Assessment in Primary Health Care

**DOI:** 10.3389/ijph.2026.1609094

**Published:** 2026-03-05

**Authors:** Saritha Shamsunder, Leela Digumarti, Bhagyalaxmi Nayak, Vasantha Dasari, Archana Mishra, Anita Kumar, Sony Nanda, Jugal Kishore, Nishi Choudhary

**Affiliations:** 1 Department of Obstetrics and Gynecology, Vardhman Mahavir Medical College and Safdarjung Hospital, New Delhi, India; 2 Department of Obstetrics and Gynecology, KIMS-ICON Hospital, Visakhapatnam, India; 3 Department of Gynecological Oncology, Acharya Harihar Post Graduate Institute of Cancer, Cuttack, Odisha, India; 4 Department Community Med, Vardhman Mahavir Medical College and Safdarjung Hospital, New Delhi, India

**Keywords:** artificial intelligence, cervical cancer screening, digital screening device, low-resource settings, screen-triage-treat, VIA-VILI

## Abstract

**Objective:**

The objective was to assess the feasibility of image-based methods for screening and triaging women in a single visit by: (i) a trained but inexperienced nurse, (ii) remote expert review via a web system, (iii) an artificial intelligence (AI) model.

**Methods:**

Sexually active, non-pregnant women (25–65 years) were screened using visual inspection method Cervical images captured with Smart Scope® CX were assessed independently by nurses, remote experts, and AI, with assessors blinded to each other. Referrals for colposcopy were based on remote expert evaluations followed by colposcopy/biopsy.

**Results:**

Among 871 women screened, AI identified 205 positives; experts identified 201. Colposcopy was performed on 69 women, 40 of them had a biopsy. Compared to histopathology, AI achieved 86.7% sensitivity, 92.0% specificity, and 90.0% accuracy (AUC = 0.894). Remote experts showed high sensitivity (86.7%) but low specificity (32%) and accuracy (52.5%).

**Conclusion:**

This study provides proof of concept for the feasibility of the AI-driven Smart Scope® CX test as a single-visit “screen-and-triage” tool in primary healthcare settings. Additionally, remote expert assessment demonstrating performance comparable to colposcopy indicates its potential as an alternative triage method in low-resource settings.

## Introduction

Current cervical cancer screening methods—including the Papanicolaou (Pap) test, human papillomavirus (HPV) testing, and visual inspection with acetic acid and Lugol’s iodine (VIA–VILI)—often require substantial clinical expertise or multiple patient visits. The World Health Organization (WHO) recommends HPV DNA testing as the primary screening method [1] and VIA–VILI in low-resource settings [2], both of which have been adopted in India [3]. However, despite the availability of these screening strategies, several challenges persist, particularly inadequate follow-up of abnormal screening results, which limits the effectiveness of cervical cancer control programs. Poor follow-up at the primary care level contributes to increased mortality and reduced quality of life [4]. Successful screening programs therefore, rely on well-functioning referral systems that ensure timely, affordable, and accessible diagnostic evaluation and treatment [5–7].

To address these gaps, a “screen–triage–treat” approach was piloted at three geographically diverse primary health centres (PHCs). During screening, trained nurses used the Smart Scope® CX (SSCX) device to capture cervical images. These images were independently assessed by (i) trained nurses, (ii) remote experts through a telemedicine platform, and (iii) an artificial intelligence (AI) model, with all assessors blinded to each other’s evaluations.

Although AI-based tools are not yet approved for independent clinical decision-making, their performance in supporting screening and triage workflows is actively being evaluated. If shown to be effective, AI-assisted assessment could support nurses in triaging women, reduce loss to follow-up, and streamline care delivery in resource-constrained settings. Findings from this pilot study will inform the design of a larger study to validate this approach.

## Methods

### Device

**FIGURE 1:**
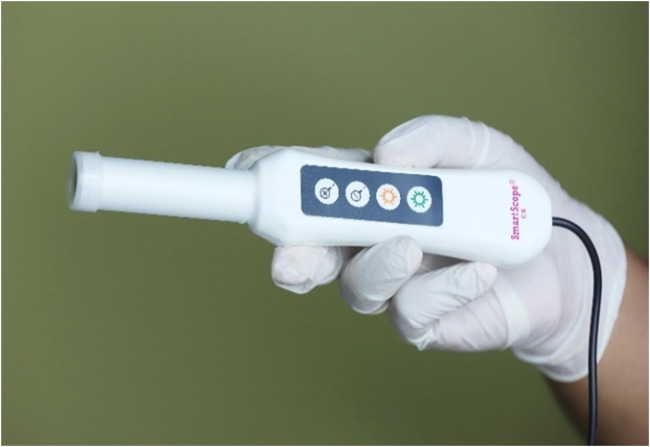
Smart Scope® CX (India, 2025)

The Smart Scope® CX (SSCX) is a portable, camera-based digital cervicoscope with ×10 magnification, specifically designed for use in low-resource or remote settings (Figure 1). Following insertion of a Cusco’s speculum, the device is introduced into the vaginal cavity to capture high-resolution images of the ectocervix. The SSCX is paired with a dedicated tablet secured with a login password and provides 6–8 h of operation on a full charge.

An integrated software platform, Net4Medix®, stores patient records and cervical images, enabling longitudinal tracking and follow-up care. All data and images are encrypted/anonymized in storage and in transit. Authentication and authorization are needed for access to any data store as per the appropriate standards.

Captured images are analysed by a proprietary cloud-based AI model, which implements an ensemble of classification and image segmentation networks. The model was trained on over 375K+ Smart Scope® images obtained from previous studies and field implementation trials involving a cross-section of the population in India and Africa. Class imbalance was addressed by augmenting the dataset of the minority class. Internal validation was performed on 40% of the dataset.

The AI model is a proprietary model developed by Periwinkle Technologies, Pune, India. It categorises images into four color-coded classes: Green—Normal; Amber—Benign conditions (e.g., cervicitis, inflammation); Brown—Probable low-grade lesion; and Red—Probable high-grade lesion or invasive cancer. The combined output of the ensemble models determines class assignment. The classification thresholds were pre-specified and remained unchanged during this study; the model was “locked” before study initiation and was not upgraded during the study trial.

### Procedures

A prospective, observational, cross-sectional, single-arm, multicentric study was conducted across three tertiary care centres and their affiliated Primary Health Centres (PHCs), following approval from the Institutional Ethics Committees (IECs) at each site and registration with the Clinical Trials Registry–India (CTRI/2023/07/055002). The study sites included: (i) KIMS-ICON Hospital, Visakhapatnam (PHC: Srinivasa Charitable Trust Clinic, Vijayanagaram) (ii) VMMC-Safdarjung Hospital, New Delhi (PHC: Urban Health Training Centre, Fatehpur Beri), and (iii) Acharya Harihar Postgraduate Institute of Cancer (AHPGIC), Cuttack (PHC: Odia Bazar).

Nurses at each PHC had at least 1 year of prior experience handling the Smart Scope® device (without AI) and assisting colposcopists during cervical cancer screening, but they had not previously made triaging decisions independently. These nurses underwent standardized training in the VIA–VILI procedure and lesion identification, which included 1 month of supervised hands-on practice under tertiary centre investigators.

### Participants

Sexually active, non-pregnant women aged 25–65 years attending gynaecology outpatient departments at the PHCs were eligible for inclusion. Exclusion criteria included a history of hysterectomy, menstruation at the time of screening, or prior treatment for cervical intraepithelial neoplasia (CIN) or cervical cancer.

### Screening and Image Assessment

Following informed consent, eligible women were screened using the standard VIA–VILI protocol [8]. Cervical images were captured using the Smart Scope® CX (SSCX) device and independently assessed by three methods: (i) trained PHC nurses, (ii) an AI-based auto-assessment tool, and (iii) remote experts located at the tertiary centres via a secure web portal. The remote experts were trained and experienced colposcopists. To avoid bias, the remote expert and the colposcopist at each centre were different individuals: the remote expert determined which patients required colposcopy, while the colposcopist decided who underwent biopsy.

The VIA–VILI procedure was performed by a trained nurse, who captured cervical images using the SSCX device. The nurse immediately documented her assessment in the Net4Medix® software preinstalled on the tablet. Upon initiating the “assess” command, the AI model generated and recorded its assessment on the server. All images were simultaneously uploaded to the cloud-based server.

Remote experts accessed the images via secure login credentials and recorded their assessments through the web portal. The software was designed to maintain blinding between the nurse, remote experts, and AI model, ensuring that no assessor had access to others’ evaluations. Remote experts completed their assessments within 2–20 days, depending on availability.

Colposcopists at tertiary centres were blinded to both AI and nurse assessments. They were informed only that women were screen-positive based on the remote expert’s assessment but were blinded to lesion stage and location. Biopsies were obtained from women who were colposcopy positive. Histopathologists were blinded to the assessments made by the AI model, remote experts, and nurses.

Screen-positive criteria included: (i) acetowhite areas on VIA or yellow lesions on VILI as assessed by nurses, (ii) brown or red indicators on AI assessment, and (iii) probable low-grade or higher findings by remote experts. A Swede score ≥1 on colposcopy was considered positive. Histopathology with CIN1+ served as the gold standard diagnostic reference.

Triaging decisions were based on the remote expert’s assessment. Women classified as screen-positive by remote experts were referred to tertiary centres for colposcopy. Biopsies were subsequently obtained from colposcopy-positive women and sent for histopathological evaluation according to standard clinical protocols.

### Statistical Analysis

All statistical analyses were performed using SPSS version 20 (IBM Corp., Chicago, IL, USA). Associations between categorical variables were evaluated using the chi-square test, with a two-sided p-value <0.05 considered statistically significant. Diagnostic performance of the screening methods was assessed by calculating sensitivity, specificity, positive predictive value (PPV), negative predictive value (NPV), and the area under the receiver operating characteristic curve (AUC), with corresponding 95% confidence intervals (CIs), positive and negative likelihood ratios (LH+ and LH-), and Pearson correlation coefficient (Pearson CC).

## Results

**FIGURE 2:**
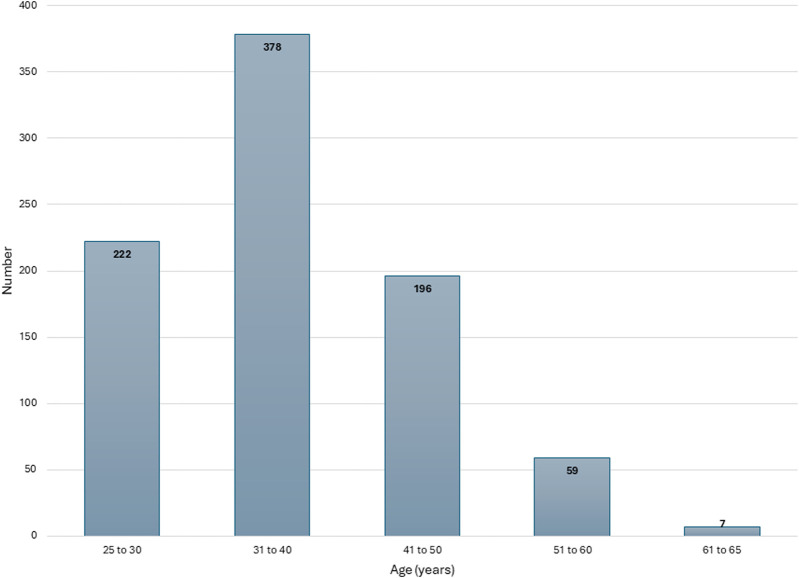
Age distribution of enrolled women (India, 2025).

Including all three PHCs, a total of 871 individuals were enrolled. PHC Odia Bazar, Cuttack, enrolled 273 women, PHC Fatehpur Beri, New Delhi 298, while PHC Srinivasa Charitable Trust Clinic, Vijayanagaram, enrolled 300 women. Figure 2 gives the number of enrolled women in each age group bracket. The majority of participants (43.4%) were aged 31–40 years, followed by those aged 25–30 years (25.8%) and 41–50 years (22.7%). Most women (73.2%) reported being married between the ages of 18 and 25 years, while 20% were married between the ages of 10 and 17 years.

Nurses, remote experts, and the AI model independently assessed the same set of cervical images captured by the SSCX during the VIA–VILI procedure. Table 1 presents the frequency distribution of enrolled women as assessed by nurses, the AI model, remote experts, colposcopists, and histopathologists.

**TABLE 1:** Percent frequency distribution of screened women on all tests (N = 871) (India, 2025).

Test	Assessment	Frequency	Percent
Nurse	Negative	819	94.03
	Positive	28	3.21
	Cannot assess	24	2.76
	Total	871	100
SSCX-AI	Green	332	38.12
	Amber	321	36.85
	Brown	32	3.67
	Red	173	19.86
	Cannot assess^a^	13	1.49
	Total	871	100
Remote expert	Normal	501	57.52
	Benign conditions	169	19.4
	Probable low-grade lesion	163	18.71
	Probable high-grade lesion	36	4.13
	Cancer	2	0.23
	Total	871	100
Colposcopy	Swede score 0	16	1.84
	Swede score 1-4	32	3.67
	Swede score 5-10	21	2.41
	Not done	802	92.08
	Total	871	100
Histopathology	Normal	3	0.34
	Benign	22	2.53
	Cervical intraepithelial neoplasia grade 1	15	1.72
	Not done	831	95.41
	Total	871	100

^a^ Image quality not good; SSCX-AI, artificial intelligence-enabled Smart Scope® test.

As shown in Table 1, of the 871 enrolled women, nurses were able to assess 847 (97.2%). For the remaining cases, nurses selected the “cannot assess” option. The AI model successfully evaluated 858 cases (98.5%), generating high-quality, sharp images, while remote experts reviewed images for all 871 women (100%). Based on image analysis, remote experts classified 201 women as screen positive. Additionally, 10 women with negative but suspicious cervical findings were also referred for colposcopy, as the remote experts opted to examine them to confirm their negative status.

**FIGURE 3:**
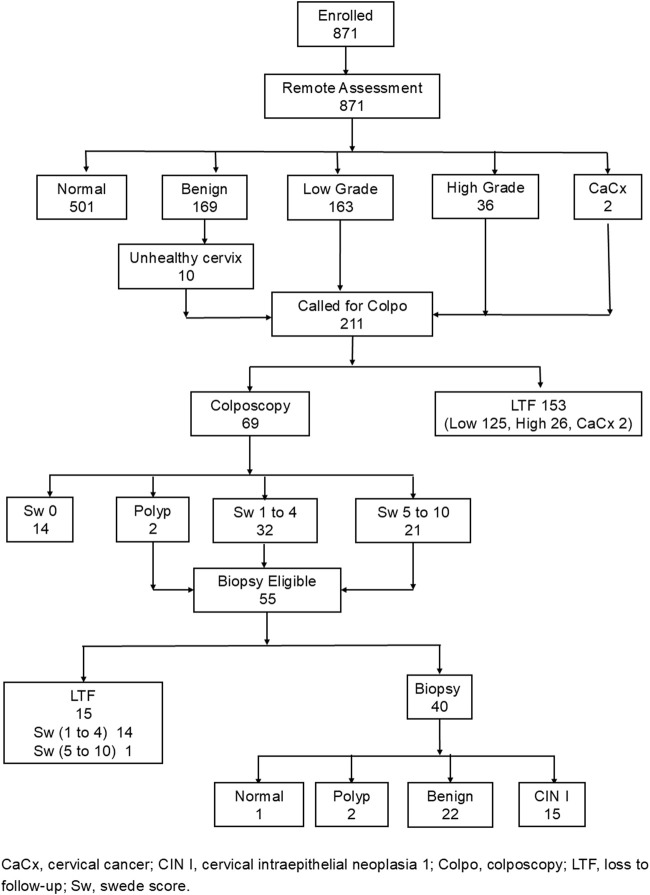
Distribution of women assessed by remote expert (N = 871) (India, 2025).

Figure 3 summarizes the number of women who were screened, triaged, referred, attended colposcopy, underwent biopsy, and were diagnosed with CIN1+ on histopathology. Of the 211 women referred for colposcopy, only 69 (32.7%) attended the higher centre, accompanied by a nurse for the follow-up visit; among these, 40 women underwent a biopsy.

Table 2 presents the correlation between histopathology and the four assessment methods. Of the 40 biopsies performed, two were identified as polyps. Histopathological examination revealed CIN I in 15 of the 40 biopsy samples. Among these, nurses missed 14 cases, while both the AI model and remote experts each missed 2 cases. Conversely, among the 25 women who were negative on histopathology, 4 were incorrectly classified as positive by nurses, 2 by the AI model, and 17 by remote experts.

**TABLE 2:** Correlation of histopathology outcome and screening results. (N = 40) (India, 2025).

Assessment method	Test result	Histopathology (40)	
		Negative (25) (Normal + Benign)	Positive (15) (CIN I)
Nurse	Negative	20	12
	Positive	4	1
	Cannot assess	1	2
SSCX-AI	Negative (Green + Amber)	23	2
	Positive (Brown + Red)	2	13
Remote expert	Negative (Normal + Benign)	8^a^	2^a^
	Positive (Low + High grade)	17	13
Colposcopy (standard)	Negative (swede score 0)	3	0
	Positive (swede score 1–10)	22	15

^a^ Though negative on remote expert assessment, as cervixes were unhealthy, they were marked for colposcopy. SSCX-AI, artificial intelligence-enabled Smart Scope® test.

The statistical analysis of the data, compared with the gold-standard histopathology, is presented in Table 3. Nurses who underwent a structured month-long training program including approximately 100 screenings, were unable to achieve adequate accuracy in image-based assessment or triage decisions. As shown in the table, the sensitivity of nurse assessment was very low (7.7%).

**TABLE 3:** Statistical parameters of screening tests against gold standard histology (India, 2025).

Parameter	Nurse value (95% CI)	SSCX-AI value (95% CI)	Remote expert Value (95% CI)
N	37	40	40
Sensitivity %	7.7 (0.0–22.2)	86.7 (69.5–100)	86.7 (69.5–100)
Specificity %	83.3 (86.4–98.2)	92 (81.4–100)	32 (13.7–50.3)
PPV %	20 (44.9–100)	86.7 (69.5–100)	43.3 (25.6–61.1)
NPV %	62.5 (45.7–79.3)	92 (81.4–100)	80 (55.2–100)
Accuracy %	56.8 (40.8–72.7)	90 (80.7–99.3)	52.5 (37.0–68.0)
AUC	0.445 (0.26–0.65)	0.894 (0.78–1.0)	0.549 (0.41–0.77)
LH+	0.46 (0.08–2.82)	10.84 (2.83–41.45)	1.27 (0.35–4.70)
LH-	1.11 (0.16–7.59)	0.145 (0.04–0.56)	0.415 (0.11–1.60)
Pearson CC	−0.125 (-0.43–0.21)	0.763 (0.59–0.87)	0.209 (−0.11–0.488)

95% CI, confidence interval; N, number of women; SSCX-AI, artificial intelligence-enabled Smart Scope® test; PPV, positive predictive value; NPV, negative predictive value; AUC, area under the curve; LH+, positive likelihood ratio; LH-, negative likelihood ratio; Pearson CC, Pearson correlation coefficient.

**FIGURE 4:**
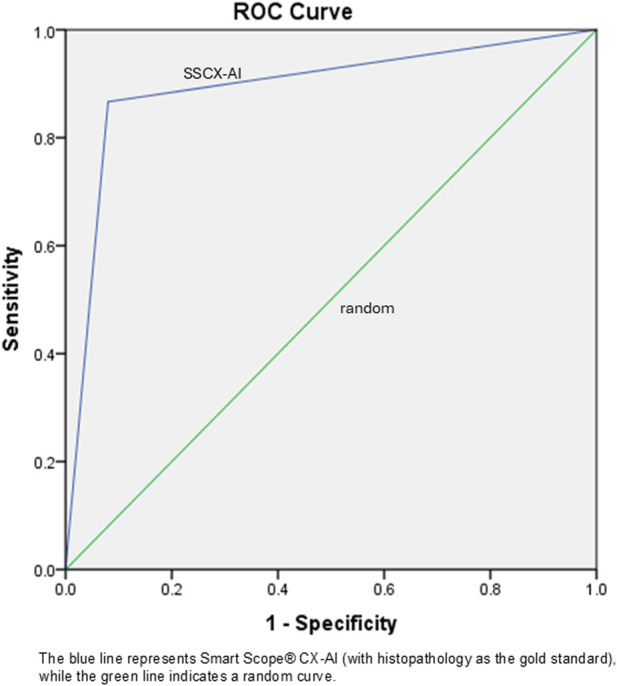
Receiver operating characteristic curve of artificial intelligence (India, 2025).

In contrast, the AI model demonstrated satisfactory performance, with a sensitivity of 86.7%, specificity of 92.0%, and overall accuracy of 90.0% (Chi-square = 24.75, p < 0.001), although it missed two true positive cases. Both the positive predictive value (PPV) and negative predictive value (NPV) of the AI model were promising for a pilot study. The area under the curve (AUC) for the AI model (Figure 4) was 0.894 (95% CI: 0.775–1.00, p < 0.001), with a positive likelihood ratio of 10.84 and a Pearson correlation coefficient of 0.787.

When the performance of the AI model was compared with remote expert analysis, it showed a specificity of 77.5% and a sensitivity of 29.5%, resulting in an overall accuracy of 66.6% (Chi-square = 4.5, p = 0.034). The preliminary estimate of concordance between AI and colposcopy was 47.8% (Pearson correlation coefficient = 0.262), while concordance with remote expert assessment was 66.5% (Pearson CC = 0.073), indicating weak agreement in both cases.

Comparing remote expert assessments with histopathology revealed high sensitivity (86.7%) but low specificity (32%) and low PPV (43.3%), reflecting a high rate of false-positive cases and resulting in overall low accuracy (52.5%). Preliminary concordance between remote expert assessments and colposcopy was 72.5% (Pearson CC = 0.302), suggesting a moderate level of agreement between the two methods.

## Discussion

The primary objective of this pilot study was to evaluate the feasibility of screening and triaging women in a single visit within PHC settings. Currently, in PHCs, screening is performed by nurses, while triage occurs at higher centres through colposcopy. This study, therefore, assessed the feasibility of image-based, immediate triage using three assessment methods. Currently, AI is not an officially approved method of assessment; thus, trained experts participated as remote experts and independently evaluated the same set of cervical images reviewed by both the AI system and the trained nurses. The remote experts’ assessments served as the triage method for determining referrals for colposcopy.

To evaluate the potential role of AI in triage, three key analyses were performed: (i) comparison of AI assessment against the gold standard histopathology, (ii) concordance between AI assessment and the currently accepted triaging method, colposcopy, and (iii) concordance between remote expert evaluation and AI assessment. Previous studies have explored the application of AI for triaging in cytology-based screening and reported promising results [9, 10]. Similarly, the present study suggests a promising performance of AI assessment, although differences exist in the screening tests and AI models used.

In a pilot study, Talathi et al. compared the Smart Scope® AI (SS-AI) with colposcopy for its application as a screening and triage tool [11] and found the SS-AI system to be comparably effective to colposcopy. In contrast, the concordance between AI and colposcopy or remote expert assessment was weak in the present study. This discrepancy may be attributed to the larger number of experts from multiple PHCs and higher enrolment in our study, compared with the single-centre, smaller sample in Talathi et al.’s study. AI has also been investigated as a triaging tool to guide biopsy decision-making, demonstrating potential to improve diagnostic accuracy and efficiency [12]. In the present study, the AI model both overestimated and missed two cases each, corresponding to an error rate of 4.9% in both directions, which is an acceptable margin for preliminary screening. The AI demonstrated an adequate positive likelihood ratio, AUC, and Pearson correlation coefficient, indicating good agreement between AI assessments and histopathology results. These findings support the feasibility of further evaluating AI in a large-scale screening study as a single-visit “screen-and-triage” tool. Implementing such a model could enable immediate treatment or timely referral to higher centres, reducing loss to follow-up and improving overall program effectiveness.

We also assessed the feasibility of using expert colposcopists for remote evaluation of digital cervical images to enable point-of-care triaging. Previous studies have demonstrated the potential of digital imaging in cervical health assessment. Shamsunder et al. [13] employed digital images for cervical screening, while Touni et al. [14] used cervicograph-captured images for remote interpretation by three experts, reporting improved VIA assessment accuracy. Millien et al. [15] applied digital images for nurse training, though quantitative outcomes were not reported. In the present study, remote experts accurately identified 13 of 15 histologically confirmed positive cases. Compared to colposcopy findings, remote image evaluation correctly classified 50 of 69 cases, yielding an accuracy of 72.5%. Remote expert assessment, like colposcopy, demonstrated high sensitivity but low specificity and low PPV, reflecting a high rate of false positives and overall low accuracy. However, the high concordance between remote expert assessments and colposcopy findings suggests that expert evaluation could serve as a viable alternative to colposcopy for triaging, particularly in settings with limited colposcopy resources. Although the original intention was to provide immediate post-screening remote expert review, this was rarely achievable due to limited expert availability. In most cases, assessments were completed approximately 1 week after image acquisition, limiting the utility of this approach for single-visit triage. Nevertheless, remote expert consultation remains valuable when the primary screener requires a second opinion.

An additional objective of this study was to assess the feasibility of nurse-led assessment using digital cervical images, without the assistance of artificial intelligence. Previous research has explored single-visit “see and treat” or “screen and treat” strategies for cervical cancer control, in which nurses not only performed screening but also managed treatment for screen-positive women. For example, Sankaranarayanan et al. [16] demonstrated that a nurse-led “see and treat” approach using cryotherapy under medical supervision was acceptable, safe, and effective in low-resource settings. Similarly, Singla et al. [17] evaluated a single-visit VIA–VILI and LEEP model at a tertiary care centre, where trained nurses conducted VIA–VILI screening and physicians performed the LEEP procedure. Their findings supported the feasibility and effectiveness of these approaches. In contrast, our study found that nurses, trained through a structured month-long program encompassing approximately 100 screenings, were unable to achieve adequate confidence in image-based assessment or triage decision-making. It is important to note that only 15 confirmed CIN I lesions were detected in this study, which may have been difficult for newly trained nurses to identify. Additional training, practical experience, ongoing mentorship, or AI assistance could potentially improve their performance. These findings highlight the challenges of implementing nurse-led screening and triage at the PHC level and underscore the need for rapid, accurate, and objective assessment methods. Integrating automated technologies can help reduce reliance on human interpretation alone and strengthen cervical cancer prevention strategies.

### Strengths and Limitations

This study has several notable strengths. First, it was a multicentre study including women from diverse ethnic backgrounds, enhancing the generalizability of the findings. Second, the entire screening and assessment process was independently carried out by nurses at the PHC level. This contrasts with the current three-stage process, in which screening is performed by a nurse at the PHC, counselling by a non-gynaecologist at the PHC, and triage by an expert colposcopist based at a tertiary centre. The study demonstrates the feasibility of task-shifting in resource-limited settings, provided structured training and continuous monitoring are implemented, potentially advancing towards the “screen and treat” model recommended by WHO. Third, triage decisions were made by remote experts based on images uploaded to a centralized server, highlighting the potential of telemedicine support in PHCs where on-site specialist availability is limited.

A major challenge encountered in the study was the high rate of loss to follow-up. Remote expert interpretations formed the basis for referral to colposcopy-guided biopsy. However, reliance on remote expert assessment introduced a delay of 10–20 days before triage decisions could be communicated. Consequently, of the 211 women referred, only 69 (32.7%) presented at a higher centre for colposcopy, resulting in 40 biopsies. Additionally, the evaluation of AI and nurse performance against the “gold standard” was limited to women pre-selected as abnormal by remote experts, rather than the entire screened population. As a pilot study, the relatively small sample size and limited histopathology data render it underpowered to detect small to moderate differences between the assessment methods. A small percentage of images (1.4%) were not assessed by the AI due to poor image quality, which could be improved through enhanced monitoring and training of nurses during image capture.

This pilot study was designed to evaluate feasibility and estimate effect sizes for a prospectively planned diagnostic accuracy study incorporating remote expert assessment and AI-based approaches as complementary tools. The results are intended to inform the design of a full-scale study in which all screened women, or a randomly selected subset, would undergo colposcopy and biopsy for outcome verification. We hypothesize that integrating AI into a single-visit screening and triage model, combined with comprehensive patient counselling, may significantly reduce loss to follow-up in resource-limited settings.

### Conclusion

This study provides proof of concept for the feasibility of the AI-driven Smart Scope® CX (SSCX) test as a single-visit “screen-and-triage” tool in primary healthcare (PHC) settings, supporting its potential role in AI-enabled public health screening programs. Additionally, the performance of remote experts, which was comparable to colposcopy, highlights their potential as an alternative triage method in low-resource settings. While both AI and remote assessment show promise, further evaluation in a large, prospectively designed diagnostic accuracy study is required to confirm their non-inferiority. In the long term, we hypothesize that healthcare workers, such as nurses, could effectively rely on complementary tools such as remote expert evaluations or AI-based tools for cervical cancer triage.
